# From Prejudice to Evidence: The Case of Rhizoma Coptidis in Singapore

**DOI:** 10.1155/2014/871720

**Published:** 2014-12-25

**Authors:** Chin Ee Ho, You Li Goh, Chang Zhang

**Affiliations:** ^1^Dongfang Hospital, Second Affiliated Hospital of Beijing University of Chinese Medicine, No. 6, District 1, Fangxingyuan, Fangzhuang, Fengtai District, Beijing 100078, China; ^2^School of Biological Sciences, Nanyang Technological University, 50 Nanyang Avenue, Singapore 639798; ^3^School of Acupuncture-Moxibustion and Tuina, Beijing University of Chinese Medicine, 11 North East Third Ring Road, Chaoyang District, Beijing 100029, China

## Abstract

Rhizoma Coptidis (RC), commonly known as *huanglian*, is a herb frequently used in Traditional Chinese Medicine (TCM) prescriptions. Known to have “*clearing damp-heat, quenching fire and counteracting poison*” properties, it was widely used in the Chinese community in Singapore. Berberine, an alkaloid isolated from RC, is known to have a wide array of therapeutic effects including antimicrobial, antineoplastic, and hepatoprotective effects. In 1978, RC was implicated in causing neonatal jaundice (NNJ) and kernicterus in neonates suffering from glucose-6-phosphate dehydrogenase (G6PD) deficiency, leading to the banning of RC and berberine in Singapore. More than three decades later, accumulating evidence-based studies pointing to the safety of RC for general public and better understanding of G6PD deficiency, the Health Sciences Authority (HSA) in Singapore reviewed and lifted the prohibition on RC and berberine, turning a brand new chapter in the history of TCM in Singapore. This paper aims to review the safety of RC and berberine, using the prohibition of use and subsequent lifting of ban on RC and berberine in Singapore as an illustration to highlight the importance of evidence-based studies in Traditional Chinese Medicine (TCM).

## 1. Introduction

With the increasing popularity in seeking complementary and alternative medicine (CAM) as a healthcare service, the prevalent use of herbal medicine as part of treatment is inevitable. Along with the rapid growth in consumption comes the pressing question on the safety of herbal medicine. While much research and investigation on the potential uses of herbal medicine has been done widely, evaluation on the safety of herbal medicines is relatively scarce. The lack of knowledge of the nature and mechanism of interaction of herbal medicines in the human body has brought about exaggerated reports and extreme measures to counter the adverse effects reported. This paper aims to review the safety of Rhizoma Coptidis (RC) and berberine, using the prohibition of use and subsequent lifting of ban on RC and berberine in Singapore as an illustration to highlight the importance of evidence-based studies in Traditional Chinese Medicine (TCM).

## 2. Rhizoma Coptidis and Berberine

RC is a commonly used TCM herb for “*clearing damp-heat, quenching fire and counteracting poison*” and is found in prescriptions for various ailments including febrile illness, hepatobiliary diseases, and gastroenteritis [1]. The earliest record of RC dates back to* Shen Nong Ben Cao Jing* (*Shen Nong's* Herbal Classic) compiled in the Han Dynasty of China, in which it was classified as a top-grade drug [2]. The prevalent use of RC as part of a TCM compound formula (*fufang* in Chinese) was cited in several renowned TCM medical compilations, namely,* Shang Han Za Bing Lun* (Treatise on Febrile and Miscellaneous Diseases),* Wai Tai Mi Yao* (Medical Secrets from Royal Library), and* Ben Cao Gang Mu* (*Compendium of Materia Medica*) [3], some of which are still in use today clinically and in evidence-based studies [4–7]. TCM formulae containing RC have also been used during pregnancy and in neonates for various conditions including neonatal jaundice (NNJ) [8].

Berberine, an alkaloid isolated from RC, was first isolated in the early nineteenth century [9]. Numerous studies on these two subjects have been conducted. Berberine and RC were also reported to have antimicrobial effects [10, 11] and are used to treat bacteria-induced diarrhea [12]. Recent area of interest is the antineoplastic effects of RC and berberine [13, 14], specifically in areas of breast cancer [15, 16], leukemia [17, 18], gastric cancer [19, 20], pancreatic cancer [21], and nasopharyngeal cancer [22]. It was also reported that RC and berberine have hepatoprotective [23], nephroprotective [24], neuroprotective [25], and cardioprotective [26] effects. Studies have also explored the effect of berberine and RC in controlling metabolic syndrome [27], hyperlipidemia [28, 29], and type II diabetes [30], highlighting berberine and RC as multifaceted drugs with immense therapeutic potential.

## 3. The Prohibition of Use of RC and Berberine in Singapore

TCM was first introduced in Singapore by the influx of Chinese immigrants in the early days as part of culture heritage and healthcare. It provided an affordable and familiar healthcare service for the new Chinese immigrants before the 1960s who found Western medical care foreign and unaffordable. TCM has since developed to become an integrative part of complementary and alternative medicine (CAM). Currently, Western Medicine serves as the mainstream of the healthcare system in Singapore [31]. Nonetheless, TCM remains the most widely used CAM in Singapore, accounting for 88% of CAM use locally [32]. Prior to its ban in 1978, RC was widely consumed as part of oral administered compound formula in Singapore. Its properties of “*clearing damp-heat, quenching fire and counteracting poison*” as described earlier were suitable to treat diseases caused by tropical weather in Singapore.

**Figure 1:**
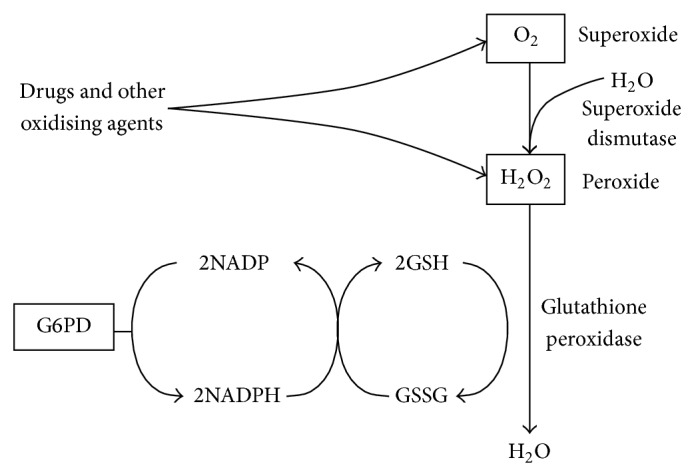
G6PD generates the NADPH which protects the erythrocytes against peroxides and superoxides generated by oxidative stresses [43].

Mass screening for G6PD deficiency in newborns has been introduced in Singapore since 1965. This is a vital move as preventive measures and effective counseling needed to be instituted early to prevent exposures to triggers [33]. Under this surveillance program, more than two decades of prevalent kernicterus was observed in Singapore. It was found that half of the babies suffering from kernicterus were suffering from deficiency of glucose-phosphate-6 dehydrogenase (G6PD) deficiency [34, 35]. G6PD deficiency is an X-linked disorder of the antioxidant homeostasis that is caused by mutations in the G6PD gene [36]. This condition currently affects about four hundred million people worldwide [37], making it the most common enzymopathy in the world. G6PD is an important enzyme that helps to protect the erythrocytes from oxidative damage. Within the restricted metabolism of erythrocytes, G6PD catalyses the first step in the hexose monophosphate pathway, converting glucose-6-phosphate to 6-phosphogluconolactone and reducing the cofactor nicotinamide-adenine dinucleotide phosphate (NADP) to NADPH. The second enzymatic step in the pathway is also associated with the reduction of NADP to NADPH (Figure 1). As G6PD is the only source of NADPH, which is essential in the protection of erythrocytes from oxidation, premature lysis of erythrocytes may occur in the absence of the enzyme [38]. In severe cases, this results in NNJ and kernicterus, causing permanent damage to the brain, resulting in mental retardation, convulsion, cerebral palsy, hearing deficit, or even death [37, 39, 40]. Triggers identified include common drugs like aspirin, methylene blue, primaquine and nitrofuran, and common environmental factors like mothballs (naphthalene), henna, and fava beans [41–46]. Infections have also been identified as trigger of hemolysis in G6PD deficient individuals [38, 47, 48]. Chinese herbal medicine containing berberine was also identified as triggers of acute hemolysis in G6PD deficient babies [49].

In the 1980s, a study in Singapore found that a high level of “indirect plasma bilirubin” was observed in local babies in general and nearly 100% were visibly jaundiced in the first week of life, compared to about 30% in Caucasian babies born in Singapore [50]. It also found that a retrospective comparison of a cohort of G6PD deficient neonates yielded a result of 22 out of 102 suffering from severe NNJ after exposure to TCM herbal medicines* in utero,* as compared to 2 out of 34 for those without. The same study also observed that exposure to mothball also triggered NNJ with a prevalence of 29 out of 100 as compared to the 20 out of 113 who were not exposed to mothballs. The authors then concluded that TCM herbal medicines, particularly RC, were the cause of severe NNJ in G6PD deficient neonates in Singapore [50]. Based on this study, the Department of Health (known as Ministry of Health now) announced the prohibition of use RC and items containing berberine in Singapore. These items have since been regulated under the Poisons Act until 2013 [31]. The prohibition had forced local TCM practitioners to need to source for substitute Chinese herbal medicine (CHM) during treatment, which affected the efficacy of the compound formula prescribed for patients.

## 4. Safety of RC and G6PD Deficiency Studies 

The implementation of this policy sparked active research and discussion in Hong Kong, Taiwan, and China, where prevalence of G6PD deficiency is pronounced and use of TCM is ubiquitous. More studies observing the safety of RC, as well as its relation to G6PD deficient individuals, were also conducted, bringing greater understanding to the safety of the herb and genetic condition.

### 4.1. *In Vivo* and* In Vitro* Studies of RC and Berberine

Several studies have supported the retrospective epidemiology findings described by Wong [50] that identified CHM, particularly RC, as the cause of NNJ in G6PD deficient neonates. A study by Ko et al. on the prooxidative effects of Chinese herbal medicine on G6PD deficient erythrocytes* in vitro* found that RC significantly reduced GSH level and increased the level of methaemoglobin in G6PD deficient blood samples, pointing to the possibility of RC as the cause of neonatal jaundice in G6PD deficient neonates [51]. A study found that chronic intraperitoneal administration of 10 and 20 mg/g of berberine daily for 1 week to adult rats resulted in a significant decrease in mean bilirubin serum protein binding, due to an* in vivo* displacement effect and a persistent elevation in steady-state serum concentrations of unbound and total bilirubin, possibly caused by inhibition of metabolism [52]. Another study by Yeung et al. yielded similar results, discovering that RC had a significant effect in displacing bilirubin from its serum protein binding as assessed by peroxidase oxidation method [53], which results in elevation of free bilirubin that can readily cross the blood brain barrier, resulting in kernicterus in neonates.

Besides the potential damage that RC may cause in G6PD deficient neonates, it was also reported that RC was known to have caused several adverse reactions including respiratory failure, extrapyramidal system reactions, severe arrhythmia, liver function injury, and even death in China [54]. Studies were done to identify the toxic constituents, as well as to evaluate the safety of RC. Ma et al. identified alkaloids to be the cause of toxicity of RC. The same study also identified berberine as the main toxic constituent in RC from both their* in vivo *studies and their* in vitro* studies, stating that the lethal dose of total extract of RC was 2.95 g/kg in mice for oral administration [55]. Another study by Yi et al. evaluated the safety of the main alkaloids from RC, namely, berberine, coptisine, palmatine, and epiberberine. This study which included cytotoxicity, acute toxicity in mice, and subchronic toxicity of RC and alkaloids in RC conducted on rats reported that the oral administration lethal dose of berberine dissolved in water was 713.57 mg/kg. The study also found that subchronic 90-day oral toxicity study of RC alkaloids and RC did not yield significant differences in clinical signs, body weight gaining, organ weight changes, urinalysis and hematological parameters, gross necropsy, and histological alterations compared to the control group. The team concluded that current recommended doses of RC alkaloids and crude RC consumption are relatively safe [56].

It is also important to note that the dosage plays a crucial role in determining the toxicity of RC. In a study evaluating the no-observed-adverse-effect level (NOAEL) and toxicity of RC in rats, urinalysis reflected a significant rise in N-acetyl-beta-glucosaminidase in male rats. However, no mortality or remarkable clinical signs were observed during the study. It was also found that RC had no adverse effects on hematology and serum chemistry. The NOAEL of RC through oral administration was ultimately found to be 667 mg/kg/day for male rats and 2000 mg/kg/day for female rats [57]. In another study by Kheir et al., it was found that median lethal doses for intravenous and intraperitoneal routes of discovery were 9.0386 and 57.6103 mg/kg, inconclusive for oral administration. The team found that an oral administration of berberine at 20.8 g/kg yielded a berberine blood concentration of 0.168 *μ*g/mL, while an increase of dosage to 41.6 g/kg yielded a berberine blood concentration of 0.432 *μ*g/mL, which resulted in mortality rate of 30%. The continual increase in dosage to 83.2 g/kg yielded similar berberine blood concentration and mortality rate as that of 41.6 g/kg, suggesting that the absorption of berberine by animal's intestine system has its own limit. The team also found that the bioavailability of berberine varies with different routes of drug administration, with the highest found in intravenous administration and the lowest in oral administration. The team concluded that dosage of berberine for oral administration at 20.8 g/kg (or a berberine blood concentration of 0.168 *μ*g/mL) is safe in mice, and the safety dosage for humans would be 2.97 g/kg of human body weight, which is much higher than the clinically recommended dosage of 15 mg berberine/kg of human body weight [58].

On the other hand, there were also skeptics who questioned the hyperbole of RC as a trigger to hemolytic jaundice in G6PD deficient individuals. A study on the influence of RC and berberine on erythrocyte osmotic fragilities of G6PD deficient rats found that a general dose of RC and berberine through oral administration did not cause hemolysis to erythrocytes of G6PD deficient rats [59]. An animal study concluded that the normal dosage of 2–5 g of RC will neither cause hemolysis of red blood cells (RBC) nor change antioxidant system and functions of RBC [60]. A retrospective Cox model analysis of 412 NNJ cases was done by Wang and Lin, establishing a relationship that points out that instead of causing NNJ, RCs seem to have a preventive effect on NNJ [61].

### 4.2. Clinical and Epidemiological Observations

Clinical and epidemiological studies have also explored the effects of RC on neonates, fetal growth, and G6PD neonates. A study by Fok et al. on 1004 mother-baby pairs found that there was no difference in the degree of jaundice between the infants born to mothers with or without antenatal consumption of herbs [62]. A cohort study by Weng et al. also found that G6PD deficient neonates are at increased risk for hyperbilirubinemia even in the nursery free from agents that can potentially cause hemolysis to G6PD deficient red cells. This study demonstrated that hyperbilirubinemia may occur spontaneously even without exogenous factors for G6PD deficient neonates [63]. Another study by Weng et al. indicated that a combination of CHM with a traditional Chinese maternal diet showed a decrease in the development of jaundice in infants as compared to those mothers who did not consume such medicines and diet [64], showing evidence against the argument of CHM causing NNJ in neonates. Another study by Chuang et al. involving 9895 pregnancies found that ingestion of RC during pregnancy had no significant adverse effect on fetal growth. However, it showed a nonsignificant slight decrease in mean birth weight and increased risk of low birth weight and small for gestational age babies if the frequency of using RC was more than 56 times [65]. Another investigation by Lin et al. found that RC was unable to aggravate jaundice of G6PD in neonates in Guangxi, which is another area with a high prevalence of G6PD deficiency [66]. A review by Valaes found no convincing epidemiological data relating Chinese herbs to hemolysis in G6PD deficient neonates, noting that the only report of NNJ in G6PD neonates occurring after consumption of CHM was that reported by Singapore [37]. Another comprehensive review from China has also pointed out the safety of RC, citing several retrospective studies on the lack of association of RC with NNJ and literature reviews on ancient TCM literatures that found no mention of RC as a prohibited CHM for pregnant woman [67].

A study conducted in Singapore that explored the organ toxicity or electrolyte imbalance in patients with chronic hematological diseases found that oral concoction containing RC was not associated with any aggravation of anemia or liver dysfunction. The same study also reviewed the fact that the composition of Chinese herbs implicated in severe NNJ described by Wong [50] was not specifically analyzed. Along with the literature reviewed, the authors concluded that, based on traditional dosage and indication, the use of RC in oral concoction is safe [1].

## 5. Lifting of the Prohibition on Berberine and RC

After more than three decades of regulation under the Poisons Act, the authorities announced the lifting of the prohibition of RC in Singapore in 2012 as suggested by the Berberine Expert Panel, after taking into account the sufficient safeguards available and the safety of berberine when used appropriately. The ban will be progressively lifted, starting from the use of Chinese propriety medicine containing berberine from January 1, 2013. In the absence of major safety issues, the Health Sciences Authority will review the possible further lifting of prohibition on Chinese herbs containing berberine by 2015 [68].

## 6. Discussion

With more studies investigating the safety of RC and the effect of RC on G6PD deficient individuals, we can better evaluate the safety of RC on normal individuals and understand the relationship between G6PD deficiency and RC. It is important to note that many factors will affect the toxicity of RC to a great extent. One such factor to be concluded from the review is the route of administration. Several studies have established the toxicity of RC through intraperitoneal and intravenous injections. However, studies exploring the toxicity of RC or berberine through oral administration have found that the recommended dosage of RC and berberine clinically is safe. This was explained by Kheir et al. through their observation that oral administration of berberine actually limited the absorption of berberine as increased dosage of oral administrated berberine yielded similar berberine blood concentration, which interestingly coincides with that of the lethal dose via intravenous administration. Such study is fundamental in evaluating the safety of RC, especially since RC is typically administered orally. Such a study can also alert clinicians that other routes of administration may not be as desired as the traditional oral route when it comes to the use of traditional herbal medicines. Another factor that affects the toxicity of RC would be the dosage. Cytotoxicity,* in vivo,* and* in vitro* tests have been done to evaluate the dosage level of RC that may cause toxicity. The outcomes vary between different tests, as these outcomes may have been affected by the solvent used as well as the line of cells and breed of animals used. Nonetheless, these tests give clinicians a good idea on the subchronic toxicity of RC and its alkaloids. However, it is also crucial for one to note that these toxicity studies of RC* in vivo* and* in vitro* merely provide us with an overview of safety of RC when used individually. One should bear in mind that RC is commonly used as one of the CHM in a compound formula, which results in complicated drug-drug interactions that may alter the toxicity of RC and its alkaloids. More research could be studied to determine the safety of RC in compound formula. Clinical studies involving oral administration of RC to pregnant mothers and infants exhibited the safety of RC in humans within recommended dosage. Although such studies seem to provide a more realistic picture of the toxicity of RC clinically, it involves ethical issues and may risk the safety of the participants, especially since such studies are exploring the safety of traditional herbal medicines. Other studies have also shown that bilirubin levels of G6PD deficient neonates may spontaneously spike even in the absence of environmental triggers, shedding some light on the spontaneity of hyperbilirubinemia in G6PD deficient neonates.

The case of banning and the subsequent lifting of prohibition on the use of RC in Singaporeserved 10 as an epic illustration of how evidence-based studies on safety of herbal medicine can eventually vindicate a useful herbal medicine. The initial prohibition of RC was perhaps a decisive act back then to control and eradicate the occurrence of kernicterus in Singapore. However, the decision was made based solely on a retrospective survey with limited knowledge to the actual mechanism and the composition of the CHM involved. Reviews have commented that there was a lack of direct causal relationship between RC and NNJ in the study that had led to the eventual prohibition of RC in Singapore. This is perhaps due to both the inadequate understanding of the TCM theories that governed the use of CHM at the point of study and the overall lack of evidence-based studies on the safety of RC. After more than three decades, studies have accumulated enough evidence to prove that the use of RC in oral administered compound formula abiding by recommended dosage and indication is relatively safe. This has subsequently led to the reversal of the policy, reflecting the important role of evidence-based studies in policy making.

With increasing acceptance and use of CHM worldwide, safety of CHM is under scrutiny. While there is extensive research investigating the possible therapeutic effects of CHM, more research should be done to evaluate the safety of CHM to safeguard the interest of its users.
